# Perception of Brazilian and Portuguese teachers about the relationship between work and physical and mental health: analysis of associated individual and work-related factors

**DOI:** 10.1590/2317-1782/e20240399en

**Published:** 2026-03-27

**Authors:** Nayara Ribeiro Gomes, Bárbara Antunes Rezende, Júlio Botelho de Souza, Alberto Freitas, Adriane Mesquita de Medeiros

**Affiliations:** 1 Programa de Pós-graduação em Ciências Fonoaudiológicas, Faculdade de Medicina, Universidade Federal de Minas Gerais – UFMG - Belo Horizonte (MG), Brasil.; 2 Centro de Investigação em Tecnologias e Serviços de Saúde – CINTESIS, Faculdade de Medicina, Universidade do Porto - Porto, Portugal.; 3 Instituto de Engenharia, Politécnico do Porto - Porto, Portugal.; 4 Programa de Pós-graduação em Saúde Pública, Faculdade de Medicina, Universidade Federal de Minas Gerais – UFMG - Belo Horizonte (MG), Brasil.

**Keywords:** Teachers, Occupational Diseases, Workplace Risks, Job Satisfaction, Occupational Health

## Abstract

**Purpose:**

To analyze the association between the negative impact of work on physical and mental health perceived by Brazilian and Portuguese teachers and its relationship with sociodemographic and work-related factors.

**Methods:**

Cross-sectional observational study using nationwide survey data from teachers. The inclusion criteria were middle school teachers from 6th to 9th grade who participated in the Teaching and Learning International Survey (TALIS). Sociodemographic variables, psychosocial factors and work-related factors, such as stress and job satisfaction, were analyzed. Descriptive analyses and univariate and multivariate logistic regression analyses for each outcome and country, setting the significance level at 5%.

**Results:**

Altogether, 5,680 teachers participated in the study. The impact of work on physical health was reported by 607 Brazilian (27.3%) and 2,061 Portuguese teachers (59.5%). Regarding mental health, 598 Brazilian teachers (27.0%) and 2,247 Portuguese teachers (64.9%) perceived this impact as being attributed to work. Factors associated with perceiving such impact included being a woman; being 40 to 59 years old; having 11 or more years in the career; not having time for personal life; being dissatisfied with work, wage, or rewards; feeling stressed; lacking support from colleagues; and work devalued by society.

**Conclusion:**

There was a statistically significant association between teachers' perceptions of the negative impact of work on health and sociodemographic characteristics (e.g., sex and age) as well as aspects related to work organization, job and salary satisfaction, stress, and social support from colleagues. Understanding these factors can help implement policies and practices to improve the teachers’ work and value them.

## INTRODUCTION

The teachers’ work is recognized as an important activity in society, as they contribute to the role of educating and training future professionals. However, scientific literature has revealed that teaching can be one of the most demanding and stressful occupations^(1,2)^.

Studies indicate that teachers have a high prevalence of common mental disorders (e.g., anxiety, depression, and stress symptoms)^(3,4)^ and physical diseases (e.g., musculoskeletal pain and voice problems)^(5,6)^_._ Such comorbidities can negatively impact their professional and personal life, causing them to be often absent^(7,8)^ and, in more severe cases, even abandon the profession^(9)^.

Besides the demands of the teaching environment, teachers are exposed to many factors whose interaction may lead to illness. These include work organization and relational and social conditions, also called psychosocial factors at work^(10)^_._

The World Health Organization has emphasized the importance of prevention and intervention approaches to manage psychosocial risks in the workplace and promote healthy work environments^(11)^. Work overload^(12)^ the lack of autonomy and participation in organizational decisions^(13)^_,_ job insecurity^(14)^, violence^(15)^, and the low social support from colleagues and management^(16,17)^ are psychosocial factors at work that can harm teachers' physical and mental health. On the other hand, it is worth highlighting that when such factors are positive, they can protect and minimize these professionals’ health problems^(18)^.

Studies indicate that professionals who express greater satisfaction with their profession perceive the work environment as favorable, have confidence in their abilities to perform tasks, and report positive emotions^(19,20)^. On the other hand, job dissatisfaction is characterized by negative feelings, which can result in professional burnout and cause depression and stress symptoms^(21)^. Therefore, it is important to understand the work-related factors to which teachers are exposed to protect their health and well-being. Hence, this study aimed to analyze the association between the negative impact of work on physical and mental health perceived by Brazilian and Portuguese teachers and its relationship with sociodemographic and work-related factors.

## METHODS

This is a quantitative, observational, analytical, cross-sectional study using nationwide survey data from. Brazilian and Portuguese middle-school teachers (6th to 9th grade) who participated in the 2018 Teaching and Learning International Survey (TALIS)^(22)^, carried out by the Organisation for Economic Co-operation and Development (OECD).

TALIS investigated teachers’ and principals’ daily practices, professional development, and working conditions. This epidemiological survey collected data in 48 countries between September 2017 and November 2018 using standardized questionnaires. It had the following exclusion criteria: teachers who also worked as principals, substitute teachers, those on long-term leave, and who taught exclusively to adults. The response rates were 94.9% in Brazil (BRA) and 92.7% in Portugal (PRT). This study used free and public access data and already anonymized, ensuring the privacy of participants and exempting it from ethical approval to Resolution Nº 510/2016 of the Brazilian National Health Council.

The explanatory variables selected for this study were sex (male and female), age (in years), length of experience as a teacher at the school where they answered the survey (in years), total career length (in years), and working hours (part-time or fulltime). The questions on psychosocial factors were assessed with 12 statements (Chart 1), which had four response options: “disagree”, “strongly disagree”, “agree”, and “strongly agree” or “not at all”, “to some extent”, “quite a bit”, and “a lot”. Data analysis categorized “disagree” and “strongly disagree” as “no”, and “agree” and “strongly agree” as “yes”.

**Chart 1:** Questions on psychosocial factors and job satisfaction used in the study (TALIS)

**Variables**	**Statements**
Actively participating in school decisions	This school provides staff with opportunities to actively participate in school decisions.
Sharing responsibilities in school issues	This school has a culture of shared responsibility for school issues.
Sharing ideas about teaching	The school staff share a common set of beliefs about teaching and learning.
Being encouraged by the school to carry out new initiatives	This school encourages staff to lead new initiatives.
Good teacher-student relationship	Teachers and students usually get along well with each other.
Receiving support from workmates	Teachers can rely on each other.
Valuing the profession	I think that the teaching profession is valued in society.
Wage satisfaction	I am satisfied with the salary I receive for my work.
Satisfaction with rewards at work	Apart from my salary, I am satisfied with the terms of my teaching contract/employment (e.g., benefits, work schedule).
Stress at work	I experience stress in my work.
Having time for personal life	My job leaves me time for my personal life.
Overall job satisfaction	All in all, I am satisfied with my job.

The response variables were the self-perception of the impact of work on physical and mental health, defined from the following statements: 1. “My job negatively impacts my physical health”; 2. “My job negatively impacts my mental health”. They had four response options, approached as twofold for data analysis, as follows: “no” for “to some extent” and “not at all” and “yes” for “a lot” and “quite a bit”.

Only cases with complete data (without missing data) were considered for analyses regarding all variables, which were initially described using absolute and relative frequency (categorical variables) and measures of central tendency and dispersion (continuous variables). The chi-square test was used to identify sociodemographic and work-related factors associated with the perceived impact of work on physical and mental health. Univariate and multivariate logistic regression analyses were further conducted separately for each outcome and country. Explanatory variables were conservatively inserted into the logistic models (p-value ≤ 0.20).

Variables with a significance level of up to 20% were considered suitable for inclusion in the multivariate model. The multivariate analysis used the forward selection method, and variables associated with the negative impact of work on physical and mental health at the 5% significance level were maintained in the final model. The magnitude of association was estimated by the odds ratio (OR) obtained from the models, considering a 95% confidence interval.

The Hosmer-Lemeshow test assessed the quality of fit of the logistic models^(23)^, verifying whether the models fit the data well. In addition to the test, pseudo-R2 values were also calculated using the Nagelkerke method. The values indicate the degree of explanatory power of the model, as follows: from 0.2 to 0.4 – a moderate relationship between the predictors and the outcome; above 0.4 – a strong relationship. All analyses were performed using RStudio software, version 1.4.1717.

## RESULTS

The sample comprised 5,680 teachers – 2,221 Brazilians and 3,459 Portuguese, mostly females (BRA: 66.0%; PRT: 70.0%), with a mean age of 42 years (±9.64) (BRA) and 48.7 years (±7.50) (PRT). The mean teaching time was 14 years (±8.99) (BRA) and 16 years (±9.85) (PRT). Part-time workload was more frequent among BRA teachers (77.0% n = 1,709), and full-time in PRT (89.0% n = 3,080).

Altogether, 607 Brazilian teachers (27.3%) and 2,061 Portuguese teachers (59.5%) reported perceiving an impact of work on their physical health - female (n=376); man (n=231) in Brazil and female (n=1,557); man (n=504) in Portugal. As for mental health, 598 Brazilian teachers (27.0%) and 2,247 Portuguese teachers (64.9%) perceived a negative impact due to work. – female (n=385); man (n=213) in Brazil and female (n=1,661); man (n=586) in Portugal. In both countries, teachers reported being generally satisfied with their work (BRA: 87.0%; PRT: 92.0%). On the other hand, 37.0% of Brazilian teachers and 88.0% of Portuguese teachers reported stress at work.

Tables 1and 2 present the descriptive statistics of the variables and their association with the perception of the negative impact of work respectively on physical and mental health per country. In both countries the two response variables (regarding physical and mental health) presented a statistically significant association with most of the investigated variables.

**Table 1:** Frequency distribution and association of sociodemographic and work-related factors according to the perception of the negative impact of work on physical health among middle-school teachers in Brazil and Portugal (TALIS)

**_Negative impact of work on physical health_**									
	_1_ **_Brazil_**				_2_ **_Portugal_**				
_Variables_	_Total n (%)_	_No n (%) = 1614 (72.7)_	_Yes n (%) = 607 (27.3)_	_p-value_ _*_		_Total n (%)_	_No n (%) = 1398 (40.4)_	_Yes n (%) = 2061 (59.6)_	_p-value*_
**_Sex_**				_0.010*_					_≤0.001*_
_Females_	_1469 (66.0)_	_1093 (68.0)_	_376 (62.0)_			_2513 (72.6)_	_956 (68.0)_	_1557 (75.0)_	
_Males_	_752 (44.0)_	_521 (32.0)_	_231 (38.0)_			_946 (27.4)_	_442 (32.0)_	_504 (25.0)_	
**_Age_**				_0.043*_					_≤0.001*_
_≤ 39 years_	_952 (43.0)_	_672 (42.0)_	_277 (46.0)_			_437 (13.0)_	_237 (17.0)_	_200 (9.8)_	
_40 to 59 years_	_1191 (54.0)_	_874 (54.0)_	_317 (52.0)_			_2796 (81.0)_	_1068 (76.0)_	_1728 (84.0)_	
_≥ 60 years_	_78 (3.0)_	_65 (4.0)_	_13 (2.1)_			_226 (6.0)_	_93 (7.0)_	_133 (6.2)_	
**_Working hours at school_**				_0.069_					_≤0.001*_
_Full-time_	_512 (23.0)_	_356 (22.0)_	_156 (26.0)_			_3080 (89.0)_	_1195 (80.0)_	_1885 (85.0)_	
_Part-time_	_1709 (77.0)_	_1258 (78.0)_	_451 (74.0)_			_379 (11.0)_	_203 (20.0)_	_176 (15.0)_	
**_Working time at school_**				_0.8_					_≤0.001*_
_≤ 5 years_	_1102 (50.0)_	_802 (50.0)_	_300 (50.0)_			_1376 (40.0)_	_611 (44.0)_	_765 (38.0)_	
_6 to 10 years_	_474 (21.0)_	_349 (22.0)_	_125 (21.0)_			_443 (13.0)_	_163 (12.0)_	_280 (13.0)_	
_≥ 11 years_	_645 (29.0)_	_463 (28.0)_	_182 (29.0)_			_1640 (47.0)_	_624 (44.0)_	_1016 (49.0)_	
**_Total career time_**				_0.3_					_≤0.001*_
_≤ 10 years_	_747 (34.0)_	_554 (34.0)_	_193 (32.0)_			_306 (9.0)_	_173 (12.0)_	_133 (7.0)_	
_11 to 20 years_	_827 (37.0)_	_585 (36.0)_	_242 (40.0)_			_1010 (29.0)_	_444 (32.0)_	_556 (27.0)_	
_≥ 21 years_	_647 (29.0)_	_475 (29.0)_	_172 (28.0)_			_2143 (62.0)_	_781 (56.0)_	_1362 (66.0)_	
**_Actively participating in school decisions_**				_≤0.001*_					_≤0.001*_
_No_	_503 (23.0)_	_299 (19.0)_	_204 (34.0)_			_905 (26.0)_	_301 (21.0)_	_604 (29.0)_	
_Yes_	_1718 (77.0)_	_1315 (81.0)_	_403 (66.0)_			_2554 (74.0)_	_1097 (79.0)_	_1457 (71.0)_	
**_Sharing responsibilities in school issues_**				_≤0.001*_					_≤0.001*_
_No_	_490 (22.0)_	_304 (19.0)_	_186 (31.0)_			_989 (29.0)_	_324 (23.0)_	_665 (32.0)_	
_Yes_	_1.731 (78.0)_	_1310 (81.0)_	_421 (69.0)_			_2470 (71.0)_	_1074 (77.0)_	_1396 (68.0)_	
**_Sharing ideas about teaching_**				_≤0.001_					_≤0.001*_
_No_	_544 (24.0)_	_318 (20.0)_	_226 (37.0)_			_1025 (30.0)_	_358 (25.0)_	_667 (32.0)_	
_Yes_	_1677 (76.0)_	_1296 (80.0)_	_381 (63.0)_			_2434 (70.0)_	_1040 (75.0)_	_1394 (68.0)_	
**_Being encouraged by the school to carry out new initiatives_**				_≤0.001*_					_≤0.001*_
_No_	_465 (21.0)_	_266 (17.0)_	_199 (33.0)_			_980 (28.0)_	_327 (23.0)_	_653 (32.0)_	
_Yes_	_1756 (79.0)_	_1348 (83.0)_	_408 (67.0)_			_2479 (72.0)_	_1071 (77.0)_	_1408 (68.0)_	
**_Good teacher-student relationship_**				_≤0.001*_					_≤0.001*_
_No_	_145 (7.0)_	_68 (4.0)_	_77 (13.0)_			_106 (3.0)_	_19 (1.4)_	_87 (4.0)_	
_Yes_	_2076 (93.0)_	_1546 (96.0)_	_530 (87.0)_			_3353 (97.0)_	_1359 (98.6)_	_1974 (96.0)_	
**_Receiving support from workmates_**				_≤0.001*_					_≤0.001*_
_No_	_255 (11.0)_	_137 (8.0)_	_118 (19.0)_			_734 (21.0)_	_196 (14.0)_	_538 (26.0)_	
_Yes_	_1966 (89.0)_	_1477 (92.0)_	_489 (81.0)_			_2725 (79.0)_	_1202 (86.0)_	_1523 (74.0)_	
**_Valuing the profession_**				_≤0.001*_					_≤0.001*_
_No_	_1989 (90.0)_	_1418 (88.0)_	_571 (94.0)_			_3133 (91.0)_	_1213 (87.0)_	_1920 (93.2)_	
_Yes_	_232 (10.0)_	_196 (12.0)_	_36 (6.0)_			_326 (9.0)_	_185 (13.0)_	_141 (6.8)_	
**_Wage satisfaction_**				_≤0.001*_					_≤0.001*_
_No_	_1816 (82.0)_	_1260 (78.0)_	_556 (91.5)_			_3124 (90.0)_	_1205 (87.0)_	_1919 (93.2)_	
_Yes_	_405 (18.0)_	_354 (22.0)_	_51 (8.5)_			_335 (10.0)_	_193 (13.0)_	_142 (6.8)_	
**_Satisfaction with rewards at work_**				_≤0.001*_					_≤0.001*_
_No_	_1078 (49.0)_	_649 (40.0)_	_429 (71.0)_			_2451 (71.0)_	_821 (59.0)_	_1630 (79.0)_	
_Yes_	_1143 (51.0)_	_965 (60.0)_	_178 (29.0)_			_1008 (29.0)_	_577 (41.0)_	_431 (21.0)_	
**_Stress at work_**				_≤0.001*_					_≤0.001*_
_No_	_1390 (63.0)_	_1248 (77.0)_	_142 (23.0)_			_428 (12.0)_	_363 (26.0)_	_65 (3.0)_	
_Yes_	_831 (37.0)_	_366 (23.0)_	_465 (77.0)_			_3031 (88.0)_	_1035 (74.0)_	_1996 (97.0)_	
**_Having time for personal life_**				_≤0.001*_					_≤0.001*_
_No_	_1391 (63.0)_	_891 (55.0)_	_500 (82.0)_			_1521 (44.0)_	_367 (26.0)_	_1154 (56.0)_	
_Yes_	_830 (37.0)_	_723 (45.0)_	_107 (18.0)_			_1938 (56.0)_	_1031 (74.0)_	_907 (44.0)_	
**_Overall job satisfaction_**				_≤0.001*_					_≤0.001*_
_No_	_292 (13.0)_	_112 (7.0)_	_180 (30.0)_			_289 (8.0)_	_40 (2.9)_	_249 (12.0)_	
_Yes_	_1929 (87.0)_	_1502 (93.0)_	_427 (70.0)_			_3170 (92.0)_	_1358 (97.1)_	_1812 (88.0)_	

Note: Valid cases were obtained after eliminating missing data in each variable:

1 Brazil: n = 2221;

2 Portugal: n = 3459

Pearson’s chi-square test

* p ≤ 0.05

**Table 2:** Frequency distribution and association of sociodemographic and work-related factors according to the perception of the negative impact of work on mental health among middle-school teachers in Brazil and Portugal (TALIS)

**_Negative impact of work on mental health_**											
	_1_ **_Brazil_**				_2_ **_Portugal_**						
**_Variables_**	_Total n (%)_	_No n (%) = 1623 (70.0)_	_Yes n (%) = 598 (27.0)_	_p-value_ _*_		_Total n (%)_		_No n (%) = 1212 (40.4)_		_Yes n (%) = 2247 (59.6)_	_p-value*_
**_Sex_**				_0.010*_							_≤0._001*
_Females_	_1469 (66.0)_	_1084 (67.0)_	_385 (64.4)_			_2513 (72.6)_	_852 (70.0)_		_1661 (73.9)_		
_Males_	_752 (44.0)_	_539 (33.0)_	_213 (35.6)_			_946 (27.4)_	_360 (30.0)_		_586 (26.1)_		
**_Age_**				_0.011*_							_0.003*_
_≤ 39 years_	_952 (43.0)_	_668 (41.0)_	_284 (47.0)_			_437 (13.0)_	_175 (14.0)_		_262 (12.0)_		
_40 to 59 years_	_1191 (54.0)_	_891 (55.0)_	_300 (50.0)_			_2796 (81.0)_	_942 (78.0)_		_1854 (83.0)_		
_≥ 60 years_	_78 (3.0)_	_64 (4.0)_	_14 (3.0)_			_226 (6.0)_	_95 (8.0)_		_131 (5.0)_		
**_Working hours at school_**				_≤0.001_							_≤0.001_
_Full-time_	_512 (23.0)_	_345 (21.0)_	_167 (28.0)_			_3080 (89.0)_	_1037 (86.0)_		_2043 (91.0)_		
_Part-time_	_1709 (77.0)_	_1278 (79.0)_	_431 (72.0)_			_379 (11.0)_	_175 (14.0)_		_204 (9.0)_		
**_Working time at school_**				_0.3_							_0.065_
_≤ 5 years_	_1102 (50.0)_	_790 (49.0)_	_312 (52.0)_			_1376 (40.0)_	_511 (42.0)_		_865 (38.5)_		
_6 to 10 years_	_474 (21.0)_	_358 (22.0)_	_116 (19.0)_			_443 (13.0)_	_140 (12.0)_		_303 (13.5)_		
_≥ 11 years_	_645 (29.0)_	_475 (29.0)_	_170 (28.0)_			_1640 (47.0)_	_561 (46.0)_		_1079 (48.0)_		
**_Total career time_**				_0.2_							_≤0.001_
_≤ 10 years_	_747 (34.0)_	_542 (33.5)_	_205 (34.3)_			_306 (9.0)_	_148 (12.0)_		_158 (7.0)_		
_11 to 20 years_	_827 (37.0)_	_591 (36.5)_	_236 (39.5)_			_1010 (29.0)_	_353 (29.0)_		_657 (29.0)_		
_≥ 21 years_	_647 (29.0)_	_490 (30.0)_	_157 (26.2)_			_2143 (62.0)_	_711 (59.0)_		_1432 (64.0)_		
**_Actively participating in school decisions_**				_≤0.001_							_≤0.001_
_No_	_503 (23.0)_	_297 (18.0)_	_206 (34.0)_			_905 (26.0)_	_245 (20.0)_		_660 (29.0)_		
_Yes_	_1718 (77.0)_	_1326 (82.0)_	_392 (66.0)_			_2554 (74.0)_	_967 (80.0)_		_1587 (71.0)_		
**_Sharing responsibilities in school issues_**				_≤0.001_							_≤0.001_
_No_	_490 (22.0)_	_299 (18.0)_	_191 (32.0)_			_989 (29.0)_	_283 (23.0)_		_706 (31.0)_		
_Yes_	_1.731 (78.0)_	_1.324 (82.0)_	_407 (68.0)_			_2470 (71.0)_	_929 (77.0)_		_1541 (69.0)_		
**_Sharing ideas about teaching_**				_≤0.001_							_≤0.001_
_No_	_544 (24.0)_	_311 (19.0)_	_233 (39.0)_			_1025 (30.0)_	_303 (25.0)_		_722 (32.0)_		
_Yes_	_1677 (76.0)_	_1312 (81.0)_	_365 (61.0)_			_2434 (70.0)_	_909 (75.0)_		_1525 (68.0)_		
**_Being encouraged by the school to carry out new initiatives_**				_≤0.001_							_≤0.001_
_No_	_465 (21.0)_	_255 (16.0)_	_210 (35.0)_			_980 (28.0)_	_275 (23.0)_		_705 (31.0)_		
_Yes_	_1756 (79.0)_	_1368 (84.0)_	_388 (65.0)_			_2479 (72.0)_	_937 (77.0)_		_1542 (69.0)_		
**_Good teacher-student relationship_**				_≤0.001_							_≤0.001_
_No_	_145 (7.0)_	_67 (4.0)_	_78 (13.0)_			_106 (3.0)_	_14 (1.0)_		_92 (4.0)_		
_Yes_	_2076 (93.0)_	_1556 (96.0)_	_520 (87.0)_			_3353 (97.0)_	_1.198 (99.0)_		_2155 (96.0)_		
**_Not receiving support from workmates_**				_≤0.001_							_≤0.001_
_No_	_255 (11.0)_	_133 (8.0)_	_122 (20.0)_			_734 (21.0)_	_164 (14.0)_		_570 (25.0)_		
_Yes_	_1966 (89.0)_	_1490 (92.0)_	_476 (80.0)_			_2725 (79.0)_	_1.048 (86.0)_		_1677 (75.0)_		
**_Valuing the profession_**				_≤0.001_							_≤0.001_
_No_	_1989 (90.0)_	_1424 (88.0)_	_565 (94.0)_			_3133 (91.0)_	_1.044 (86.0)_		_2089 (93.0)_		
_Yes_	_232 (10.0)_	_199 (12.0)_	_33 (6.0)_			_326 (9.0)_	_168 (14.0)_		_158 (7.0)_		
**_Wage satisfaction_**				_≤0.001_							_≤0.001_
_No_	_1816 (82.0)_	_1264 (78.0)_	_552 (92.0)_			_3124 (90.0)_	_1.037 (86.0)_		_2087 (93.0)_		
_Yes_	_405 (18.0)_	_359 (22.0)_	_46 (8.0)_			_335 (10.0)_	_175 (14.0)_		_160 (7.0)_		
**_Satisfaction with rewards at work_**				_≤0.001_							_≤0.001_
_No_	_1078 (49.0)_	_631 (39.0)_	_447 (75.0)_			_2451 (71.0)_	_696 (57.0)_		_1755 (78.0)_		
_Yes_	_1143 (51.0)_	_992 (61.0)_	_151 (25.0)_			_1008 (29.0)_	_516 (43.0)_		_492 (22.0)_		
**_Stress at work_**				_≤0.001_							_≤0.001_
_No_	_1390 (63.0)_	_1283 (79.0)_	_107 (18.0)_			_428 (12.0)_	_367 (30.0)_		_61 (3.0)_		
_Yes_	_831 (37.0)_	_340 (21.0)_	_491 (82.0)_			_3031 (88.0)_	_845 (70.0)_		_2186 (97.0)_		
**_Having time for personal life_**				_≤0.001_							_≤0.001_
_No_	_1391 (63.0)_	_901 (56.0)_	_490 (82.0)_			_1521 (44.0)_	_294 (24.0)_		_1227 (55.0)_		
_Yes_	_830 (37.0)_	_722 (44.0)_	_108 (18.0)_			_1938 (56.0)_	_918 (76.0)_		_1020 (45.0)_		
**_Overall job satisfaction_**				_≤0.001_							_≤0.001_
_No_	_292 (13.0)_	_101 (6.0)_	_191 (32.0)_			_289 (8.0)_	_24 (2.0)_		_265 (12.0)_		
_Yes_	_1929 (87.0)_	_1522 (94.0)_	_407 (68.0)_			_3170 (92.0)_	_1.188 (98.0)_		_1982 (88.0)_		

Note: Valid cases were obtained after eliminating missing data in each variable:

1 Brazil: n = 2221;

2 Portugal: n = 3459

Pearson’s chi-square test

* p ≤ 0.05

In Brazil, working hours, time working at school, and total career time had no statistical significance with physical health. Also, time working at school did not seem to influence the negative impact of work on mental health among Brazilian teachers. Likewise, in Portugal, time working at school had no statistical significance with the impact on mental health.

The final multivariate model shows that the odds of Brazilian teachers reporting a negative impact of work on physical health increased as they reported being a female (OR = 1.50 95CI [1.18–1.91]), not having time for personal life (OR = 2.48 95CI [1.90– 3.24]), and being generally dissatisfied with work (OR = 2.36 95CI [1.74–3.22]), stressed at work (OR = 8.31 CI95 [6.60–10.53]), dissatisfied with salary (OR = 1.51 95CI [1.05–2.19]), and dissatisfied with job rewards (OR = 1.79 95CI [1.40–2.30]) (Table 3).

**Table 3:** Final model of factors associated with the negative impact of work on physical health perceived by teachers – Brazil and Portugal (TALIS)

	**Brazil**		**Portugal**	
**Variables**	OR (95CI)	p-value	OR (95CI)	p-value
**Sex**				
Males	1		-	-
Females	1.50 (1.18 – 1.91)	≤0.001*		
**Total career time**				
≤ 10 years	-	-	1	
11 to 20 years			1.28 (0.94 – 1.74)	0.122
≥ 21 years			1.63 (1.18 – 2.26)	0.003*
**Age**				
≤ 39 years	-	-	1	
40 to 59 years			1.46 (1.11 – 1.92)	0.007*
≥ 60 years			1.44 (0.95 – 2.20)	0.086
**Having time for personal life**				
Yes	1		1	
No	2.48 (1.90 – 3.24)	**≤**0.001*	2.67 (2.28 – 3.14)	≤0.001*
**Overall job satisfaction**				
Yes	1		1	
No	2.36 (1.74 – 3.22)	≤0.001*	2.69 (1.90 – 3.91)	≤0.001*
**Stress at work**				
Yes	1		1	
No	8.31 (6.60 – 10.53)	≤0.001*	7.88 (5.96 – 10.59)	≤0.001*
**Receiving support from workmates**				
Yes	-		1	
No			1.73 (1.42 – 2.12)	≤0.001*
**Wage satisfaction**				
Yes	1		-	-
No	1.51 (1.05 – 2.19)	0.028*		
**Satisfaction with rewards at work**				
Yes	1		1	
No	1.79 (1.40 – 2.30)	≤0.001*	1.67 (1.41 – 1.98)	≤0.001*
**Being valued by society**				
Yes	-	**-**	1	
No			1.49 (1.14 – 1.94)	0.003*
Hosmer-Lemeshow test	**3.94 (p = 0.86)**		5.06 (p = 0.75)	
Pseudo-R2 (Nagelkerke)	0.40		0.28	

* p = ≤ 0.05

Caption: OR = odds ratio; CI = confidence interval

In Portugal, this impact increased as teachers reported not having time for personal life (OR = 2.67 95CI [2.28 – 3.14]), being generally dissatisfied with work (OR = 2.69 95CI [1.90 – 3.91]), stressed at work (OR = 7.88 95CI [5.96 – 10.59]), not receiving support from colleagues (OR = 1.73 95CI [1.42 – 2.12]), being dissatisfied with job rewards (OR = 1.67 95CI [1.41 – 1.98]), profession devaluation (OR = 1.49 95CI [1.14 – 1.94]), total career time ≥ 21 years (OR= 1.63 95CI [1.18 – 2.26]), and being 40-59 years old (OR = 1.46 95CI [1.11 – 1.92]) (Table 3).

Results regarding mental health indicated that the negative impact of work increased among Brazilian teachers who reported not having time for personal life (OR = 2.17 95CI [1.64 – 2.88]), being generally dissatisfied with work (OR = 2.80 95CI [2.02 – 3.91]), stressed at work (OR = 12.86 95CI [10.01 – 16.64]), not receiving support from colleagues (OR = 1.87 95CI [1.31 – 2.66]), and being dissatisfied with job rewards (OR = 2.62 95CI [2.03 – 3.40]) (Table 4).

**Table 4:** Final model of factors associated with the negative impact of work on mental health perceived by teachers – Brazil and Portugal (TALIS)

	**Brazil**		**Portugal**	
**Variables**	OR (95CI)	p-value	OR (95CI)	p-value
**Total career time**				
≤ 10 years	-	-	1	
11 to 20 years			1.41 (1.03 – 1.92)	0.031*
≥ 21 years			1.41 (1.04 – 1.92)	0.026*
**Having time for personal life**				
Yes	1		1	
No	2.17 (1.64 – 2.88)	≤0.001*	2.78 (2.34 – 3.30)	≤0.001*
**Working hours at school**				
Part-time	-		1	
Full-time			1.35 (1.03 – 1.78)	0.030*
**Overall job satisfaction**				
Yes	1		1	
No	2.80 (2.02 – 3.91)	≤0.001*	3.72 (2.44 – 5.93)	≤0.001*
**Stress at work**				
Yes	1		1	
No	12.86 (10.01 – 16.64)	≤0.001*	11.59 (8.69 – 15.70)	≤0.001*
**Receiving support from workmates**				
Yes	1		1	
No	1.87 (1.31 – 2.66)	≤0.001*	1.71 (1.38 – 2.13)	≤0.001*
**Satisfaction with rewards at work**				
Yes	1		1	
No	2.62 (2.03 – 3.40)	≤0.001*	1.63 (1.37 – 1.94)	≤0.001*
**Valuing the profession**				
Yes	-	**-**	1	
No			1.47 (1.12 – 1.92)	0.006*
Hosmer-Lemeshow test	**3.82 (p = 0.70)**		12.9 (p = 0.16)	
Pseudo-R2 (Nagelkerke)	0.49		0.32	

Caption: OR = odds ratio; CI = confidence interval

* p = ≤ 0.05

As for Portuguese teachers, this impact increased as they reported not having time for personal life (OR = 2.78 95CI [2.34 – 3.30]), being generally dissatisfied with work (OR = 3.72 95CI [2.44 – 5.93]), stressed at work (OR = 11.59 95CI [8.69 – 15.70]), not receiving support from colleagues (OR = 1.71 95CI [1.38 – 2.13]), being dissatisfied with job rewards (OR = 1.63 95CI [1.37 – 1.94]), profession devaluation (OR = 1.49 95CI [1.14 – 1.94]), and total career time from 11 to 20 years (OR = 1.41 95CI [1.03 – 1.92]) and ≥ 21 years (OR = 1.41 95CI [1.04 – 1.92]) (Table 4).

The model fits were good. The degree of explainability had a moderate relationship in the multivariate model for Portugal (Pseudo-R2 >0.4) and a strong relationship in the model for Brazil (Pseudo-R^2^ between 0.2 and 0.4).

## DISCUSSION

This research analyzed the individual and work-related factors associated with the perception of the negative impact of work on the physical and mental health of Brazilian and Portuguese teachers. Approximately a quarter of Brazilian teachers reported that work impacted both their physical and mental health. This perception was reported by more than half of Portuguese teachers.

Teachers’ health and professional well-being have been emphasized in national and international policies and organizations, as evidenced by OECD 2018^(22)^ and Eurydice 2021 data^(24)^. These reports reveal that organizational conditions and the work environment can trigger illnesses when considered sources of negative experiences, causing physical and emotional exhaustion, significantly affecting the professionals’ health.

The results revealed that being a woman and being dissatisfied with their salary negatively impacted the physical health of Brazilian teachers. Studies^(25,26)^ revealed that women tend to balance multiple roles, including professional demands and home activities. This is because women are generally and predominantly responsible for home chores and family subsistence, with unpaid activities. Such accumulated tasks can cause work overload and make it difficult to prioritize self-care over health, increasing cases of illness.

Wage and labor division inequalities are another factor to consider regarding sex^(27,28)^. In the context of education, women still occupy positions more directly related to pedagogy, while men work in sectors focused on management and with better pay. Even among male and female teachers, burnout and stress factors tend to be more present in women's daily lives^(25,26)^.

According to OECD data^(23)^, Brazilian teachers' wages are among the lowest in developing countries. Hence, teachers take on more intense workloads to increase their income, often working in multiple schools and double shifts. This increase in work hours and workload may further worsen their health problems^(26)^. The present study showed that Portuguese teachers over 40 years old and with a career of 21 or more years perceived a negative impact of work on their physical health, and those with more than 11 years of career reported an impact on their mental health. This finding corroborates the portuguese study^(29)^, in which public elementary school teachers aged 56 to 70 years and with a longer career had lower professional well-being and faced more health problems impacted by occupational demands and challenges. The present study found that mental health suffered greater impact from shorter teaching careers, in contrast with physical health. This negative perception of work at an early age may attract and maintain fewer young people in the teaching career.

Younger teachers with a shorter career and part-time work predominated in Brazil, although these factors were not associated with the negative impact of work on health. Brazilian research^(30,31)^ report that teachers leave the profession early due to illness, absenteeism, or giving up their career. Some other factors can also contribute to this scenario, including unfavorable working conditions, low wages, and excessive workload.

The lack of statistical significance between Brazilian teachers’ age and length of service and the impact on their health may be associated with the healthy worker effect – i.e., that only those fit to work are effectively teaching. Teacher turnover, whether for personal or occupational reasons, is another factor leading many of them to leave the profession earlier than desired^(32,33)^. The perception that society did not value their profession negatively impacted the physical and mental health of Portuguese teachers alone. This devaluation was reported by 90.7% of these participants. Data released by the European Commission^(24)^ on teacher training indicate apparent job satisfaction, whereas teachers report feeling undervalued. A study reinforces that lack of professional recognition and social pressure can contribute to stress and job dissatisfaction, negatively affecting teachers' mental health^(34)^.

In the case of Brazil, the 89.5% of teachers who reported feeling undervalued also stood out. However, no significant associations were found in this study between this variable and negative impacts on the health of Brazilian teachers. It is worth noting that valuing the profession is extremely important to maintain teachers' well-being and promote their professional and personal fulfillment.

The Law on Educational System Bases (LBSE)^(35)^, which establishes the general principles of education in Portugal, highlights the importance of teachers and recognizes their fundamental role in the educational process, emphasizing the need to value and qualify the teaching profession. However, this value is rather a social than an individual responsibility, aiming to ensure social and cultural recognition of the role of teachers. In Brazil, the goals of the National Education Plan (PNE)^(36)^ for the last 10 years (2014-2024) include the appreciation of education professionals and strategies focused on continuing education, guaranteed working conditions, and progressively updated minimum wages.

Portuguese teachers reported a greater negative impact of work on their physical and mental health than Brazilian ones. Research^(37,38)^ indicates that, although both countries share historical and linguistic ties, there are significant differences in educational policies and teachers' working conditions. In Brazil, teachers face challenges related to remuneration and working conditions. Incorporating this comparative perspective can offer a more comprehensive understanding of the dynamics that affect teachers' well-being in distinct cultural contexts.

However, there were similarities regarding the factors associated with both outcomes. Hence, not having time for personal life was a relevant factor for the perception of the negative impact of work on teachers' health in both countries. This corroborates the literature, which mentions the imbalance between work and personal life as a phenomenon detrimental to teachers' health, causing emotional distress and discomfort among workers^(39)^ and the negative relationship with anxiety and depression^(40)^. Not having time for personal life indicates less time for rest and leisure with greater physical and mental overload for teachers.

Reported job and job reward dissatisfaction in this study, regardless of salary, also increased the odds of perceiving a negative impact of work on health in both countries. A review study^(41)^ found an association between low levels of job satisfaction and problems related to common mental disorders such as depression and anxiety. Similar results were found in the Brazilian population-based study^(21)^, which found a prevalence of burnout syndrome approximately three times higher in teachers who reported dissatisfaction with their work.

Research^(41)^ also confirms that being satisfied with one's job reduces physical symptoms such as headaches, stomachaches, and palpitations. The study results also reinforce the importance of satisfaction with teaching work in preventing stress and promoting health and well-being^(42)^. A study^(43)^ showed that teachers' job satisfaction regarding benefits and rewards was significantly associated with well-being at work. Likewise, being satisfied with one's job may be attributed to the positive effect on general well-being and self-perception of health conditions^(15)^.

Our findings demonstrated that feeling stressed at work was associated with both outcomes for Brazil and Portugal. This was the factor with the highest strength of association (OR), especially for the negative impact on mental health. Research has shown that work-related stress can trigger a series of negative physical reactions, including emotional exhaustion and illnesses related to common mental disorders.

Specifically in the educational context, teachers experience stress when facing significant difficulties in dealing with the various demands inherent to their profession^(2)^. OECD data^(23)^ show that the main sources of stress at work among Portuguese teachers are not directly related to essential teaching tasks, but rather to administrative tasks, responsibility for student outcomes, and demands from educational authorities. Knowing the causal effects of stress is essential to suggest coping strategies and promote healthy work environments to protect workers' health.

Our findings revealed some of these possible factors – e.g., not having time for personal life, as previously discussed, and not receiving support from colleagues –, which were associated with the study outcomes, especially the impact on mental health. This result corroborates research that indicates aspects related to teachers' illness significantly associated with low social support^(44,45)^. A study^(44)^ pointed out that teachers’ poor mental health is significantly associated with high work demands and low social support. Other research^(45)^ also states that psychosocial factors and depression are significant predictors of musculoskeletal pain in teachers.

Positive effects on self-perceived social support in the work environment can also be found in studies on the reduction of stress, depression, and somatic diseases^(16,46)^. Therefore, social support is essential to promote a healthy work environment and provide emotional support to teachers. This highlights the need to ensure practices aiming to improve interpersonal relationships and develop support networks within educational institutions.

In short, our results provide important evidence on the factors associated with teachers' health. Furthermore, the results reinforce the advances and possibilities for understanding important teaching-related aspects. Knowing these factors can help implement policies and practices to improve work organization and support teachers' well-being.

This study has some limitations. Since it approached a secondary database with predefined questions, the study could not identify factors of the physical work environment possibly related to the study outcomes. Also, cultural, political, and socioeconomic differences may affect the perception of the negative impact of work among Brazilian and Portuguese teachers’ physical and mental health. Local contexts at the time of data collection may have interfered with their responses. It is suggested that new studies with other designs and qualitative data could be explored for a greater understanding of the educational system, considering the cultural and socioeconomic differences of each country.

Despite its limitations, this study showed the possibility of using the OECD database to monitor aspects investigated in the TALIS survey over the years and compare data from different countries. Previous versions of the report did not investigate health related aspects. Thus, the inclusion of physical and mental health data in 2018 was greatly important to expand the 2008 and 2013 surveys, focused on educational aspects.

## CONCLUSION

A substantial share of Brazilian and Portuguese teachers reported negative impacts of work on their physical and mental health. Portuguese teachers reported a considerably higher impact on both, physical and mental health, compared to their Brazilian counterparts. For both countries one or both outcomes were statistically significantly associated with being a female; being 40 to 59 years old; having a total career time of more than 11 years; lacking time for personal life; being generally dissatisfied with work, wage, and rewards; being stressed; lacking social support from colleagues; and devaluation of work by society.

The evidence found in this study shows the need to ensure that teachers have adequate work organization conditions to carry out their profession. Hence, there is a need for time management strategies, social support programs, actions aimed at professional development, and partnerships between health professionals and schools to monitor and promote teachers’ physical and mental health and provide adequate support in treatment, when necessary.
